# Client perceptions of the mental health engagement network: a qualitative analysis of an electronic personal health record

**DOI:** 10.1186/s12888-015-0614-7

**Published:** 2015-10-14

**Authors:** Cheryl Forchuk, Jeffrey P. Reiss, Tony O’Regan, Paige Ethridge, Lorie Donelle, Abraham Rudnick

**Affiliations:** 1 Lawson Health Research Institute, 750 Baseline Road East, London, ON Canada; 2 Arthur Labatt Family School of Nursing, Faculty of Health Sciences, Western University, London, ON Canada; 3 Department of Psychiatry, Schulich School of Medicine and Dentistry Western University, London, ON Canada; 4 Mental Healthcare Program, London Health Sciences Centre, London, ON Canada; 5 School of Health Studies, Faculty of Science, Western University, London, ON Canada; 6 Department of Psychiatry, University of British Columbia, Vancouver, BC Canada; 7 Mental Health and Substance Use Services, Vancouver Island Health Authority, Victoria, BC Canada

**Keywords:** Qualitative analysis, Personal health record, Smartphones, Tablets, Mood disorders, Psychotic disorders, Mental illness

## Abstract

**Background:**

Information technologies such as websites, mobile phone applications, and virtual reality programs have been shown to deliver innovative and effective treatments for mental illness. Much of the research studying electronic mental health interventions focuses on symptom reduction; however, to facilitate the implementation of electronic interventions in usual mental health care, it is also important to investigate the perceptions of clients who will be using the technologies. To this end, a qualitative analysis of focus group discussions regarding the Mental Health Engagement Network, a web-based personal health record and smartphone intervention, is presented here.

**Methods:**

Individuals living in the community with a mood or psychotic disorder (*n* = 394) were provided with a smartphone and access to an electronic personal health record, the Lawson SMART Record, for 12 to 18 months to manage their mental health. This study employed a delayed-implementation design and obtained both quantitative and qualitative data through individual interviews and focus group sessions. Participants had the opportunity to participate in voluntary focus group sessions at three points throughout the study to discuss their perceptions of the technologies. Qualitative data from 95 focus group participants were analysed using a thematic analysis.

**Results:**

Four overarching themes emerged from focus group discussions: 1) Versatile functionality of the Lawson SMART Record and smartphone facilitated use; 2) Aspects of the technologies as barriers to use; 3) Use of the Mental health Engagement Network technologies resulted in perceived positive outcomes; 4) Future enhancement of the Lawson SMART Record and intervention is recommended.

**Discussion:**

These qualitative data provide a valuable contribution to the understanding of how smarttechnologies can be integrated into usual mental health care. Smartphones are extremely portable andcommonplace in society. Therefore, clients can use these devices to manage and track mental health issuesin any place at almost any time without feeling stigmatized.

**Conclusions:**

Assessing clients’ perspectives regarding the use of smart technologies in mental health care provides an invaluable addition to the current literature. Qualitative findings support the feasibility of implementing a smartphone and electronic personal health record intervention with individuals who are living in the community and experiencing a mental illness, and provide considerations for future development and implementation.

## Background

Health care services have benefited from improved accessibility, quality, and efficiency afforded by developments in information technologies. Successful interventions in the mental health field have used technologies such as virtual reality [1], webpages [2], and smartphones [3]. These technologies have been used to complement treatment of a wide range of illnesses, for example, schizophrenia [4], bipolar disorder [2], and social phobia [5]. Health care technologies compatible with mobile phones are particularly impactful due to the ubiquitous and portable nature of these devices. While a variety of technologies have proven successful in improving mental health outcomes and treatment accessibility [6], the importance of client input regarding the experience of using an intervention and suggestions for future development cannot be overstated [7]. For this reason, qualitative data regarding participants’ perspectives is an essential part of health technology research. The Mental Health Engagement Network (MHEN) study sought to explore the use of web and mobile technologies in the care of individuals experiencing mood or psychotic disorders [8–11].

This study implemented a web-based personal health record accessible on smartphones for clients and tablets for care providers. The purpose was to facilitate communication between clients and care providers, to assist clients with self-health monitoring and health management, and to assist care providers with their role in supporting their clients’ in these activities. The present study is a qualitative analysis of data collected in the MHEN study in order to elaborate upon clients’ experiences and the outcomes of introducing these technologies. In particular clients’ perceptions of the facilitators and barriers to adoption of the MHEN technologies are considered. This analysis, including clients’ recommendations and suggestions will help to inform future development and implementation of similar mental health care technologies.

### Literature review

Literature describing the use of technology in mental health care is growing. As such, a number of quantitative analyses of clients’ current use of technology, as well as their willingness to use technology in their care, have been reported. Many of these studies have indicated that a majority of mental health clients are willing and able to integrate mobile technologies into their care and that mental health service providers believe clients will benefit from such technologies [4, 12, 13]. In addition to this evidence, there is an abundance of quantitative research reporting the efficacy of specific mental health interventions. Mobile phone interventions have been found to reduce the severity of hallucinations and increase social interactions [14], decrease feelings of depression and general distress [15], and reduce psychotic symptoms and general psychopathology [16].

Studies have used quantitative measures to assess participants’ perceptions of healthcare technologies. An online intervention for psychosis was found to make participants feel safe, empowered, and more socially connected [17]. Mental health mobile phone applications have also been reported as helpful to clients, to doctors in better understanding their clients, and as recommended for use by peers [18, 19]. A specific type of electronic intervention that has garnered attention in recent years is the personal health record (PHR). PHRs have been shown to promote self-management of health and to be acceptable to use among individuals experiencing mental illnesses [20–22].

Evidently, many mental health technologies have been implemented, evaluated, and proven beneficial. Qualitative literature regarding the use of technologies in mental health care is scarce; yet, this type of data remains of vital importance to the development of the field and to the improvement of patient care. Several studies stress that well designed quantitative and qualitative research can be complementary, providing a nuanced understanding to the research issue [23, 24].

The importance of qualitative data is exemplified in Gately, Rogers, Kirk, and McNally’s [25] qualitative meta-synthesis of studies using home health technologies for management of chronic illnesses. This meta-analysis included studies focusing on chronic physical illnesses, such as kidney disease and diabetes, rather than mental health issues, but the emergent themes remain informative for mental health technology research. The authors identified several themes that are relevant to the use of mobile devices in mental health service delivery including, “The reconstruction of identity”, “Coming to terms with living a technology-assisted life”, and “Usability of devices”. Participants also identified a good fit between their lifestyle and use of the technology as an important factor in determining their perceptions of the technology. User friendly technologies and participants’ competency with technology were both important factors in the acceptance of interventions [25].

One of the few studies that completed a qualitative analysis of data from a mobile phone-based intervention with individuals experiencing severe mental illness revealed important themes and issues related to adoption of the technology [26, 27]. This study compared a smartphone application built into the device and a short message service (SMS) text-only implementation of a diagnostic assessment tool for individuals (*n* = 24) with schizophrenia or schizoaffective disorder. Three central themes emerged through qualitative analysis: 1. Appeal of usability and familiarity—Participants voiced a preference for an application that was streamlined (i.e., fast and easy to use). 2. Acceptability, validity, and integration into domestic routines—Participants also noted that using a mobile phone is common in society and therefore non-stigmatizing 3. Perceived impact on clinical care—Interestingly, some clients suggested that discussing sensitive or embarrassing issues was easier using a mobile device, rather than in a face-to-face conversation. It is important to note, however, that not all responses were positive, with some participants identifying that focusing on symptoms made them preoccupied with their thoughts or reminded them of past phases of acute illness [27]. This finding mirrors an earlier thematic analysis completed by Nicholas and colleagues, which reported that one reason for non-adherence to an intervention was a disinclination to think about one’s illness [2].

Another study with a small sample (*n* = 8) of former inpatients with comorbid psychiatric illness and substance abuse problems reported similar qualitative findings [28]. Participants in the study used their personal mobile devices and were given prepaid phone cards to communicate with a staff member with whom they had a well-established relationship. Qualitative (semi-structured) interviews were completed with clients (*n* = 8) and staff members (*n* = 8) and, again, the authors reported three major themes: 1. Overall use of SMS - Approximately half of the SMS messages sent by participants were related to drug or psychiatric issues. 2. Impression management—Similar to the finding reported by Palmier-Claus and colleagues [27], participants found that sending messages through mobile devices prevented them from feeling embarrassed and allowed them to present a self-image with which they felt comfortable 3. Perceived presence—Participants described feeling more connected to another person, even when they were not actively using mobile communication. One participant described feeling as if there was “a permanently outreached hand”.

While limited, these qualitative analyses exploring use of technology in health care are informative for understanding clients’ perceptions of using technology in their care, as well as key issues to consider for future development of mobile mental health technologies. Importantly, emergent themes, such as usability, were similar across studies. However, these studies are lacking in some significant ways. The sample sizes in both of the mental health-related studies are relatively small, with samples of 24 and 16 for the studies completed by Ainsworth and colleagues [26], and Bjerke and colleagues [28], respectively. Second, these studies were completed over relatively short periods of time. The study by Ainsworth and colleagues, for example only implemented each technology for one week [26]. Participants may have been able to provide initial impressions and predict how they would use the technology over a longer period of time, but data regarding how perceptions of the technology change over time would also be informative. The MHEN study addressed both of these issues by implementing a smartphone intervention for 12 to 18 months in a larger sample. Thus, qualitative analysis of data from the MHEN study will constitute an important addition to the literature relating to using smart technologies in mental health service delivery, and will inform future development and implementation.

### The primary study

The MHEN study provided mental health clients with a web-based PHR referred to as the Lawson SMART Record (LSR). The LSR operates from the TELUS health space^TM^ platform which is powered by Microsoft^TM^ Health Vault^TM^. An interdisciplinary team of researchers, health care providers, and mental health clients developed the LSR in collaboration with information technology experts. The LSR has several important functions allowing for clients to self-manage mental health issues and connect with their care providers; for example, secure messaging between clients and care providers, a mood monitor, a health journal, and access to a personal crisis plan. During the study, both participating care providers and clients received a LSR account. Care providers also received an internet enabled tablet (iPad), while clients received an internet enabled smartphone (iPhone 4S) on which to access the LSR. At the end of the study, the service plan on all devices was deactivated but care providers and clients were able to keep their devices.

The clients’ smartphones and care providers’ iPads were preinstalled with the Lawson SMART record. All study participants were required to attend half-day training sessions prior to receiving their devices. Training sessions were designed in collaboration with consumers, key stakeholders, and mental health professionals and with community and industry partners who possessed technology or training expertise. Training sessions included an instructional demonstration and overview component as well as hands-on opportunities to explore the applications and features of both the LSR and the smartphone or tablet device itself. Each individual’s comfort and literacy with the technology was established at this time, with the opportunity to ask questions and receive technical support. A set of materials was also provided for participants to take away which included screen shots and written guidance. Additionally, weekly drop-in sessions were provided for those who wished to extend their knowledge or who were experiencing any technical difficulties. The research coordinator was the designated contact person for participants and in exceptional circumstances arranged for at-home visits (e.g., where transportation or mobility issues existed). Furthermore, participants were kept informed about relevant resources, future drop-in sessions or other announcements through the creation of a blog.

It was anticipated that most accessibility to the LSR would be through the devices provided but as the LSR was a web-based application it could be accessed on any internet enable device and thereby accommodate personal choices. This functionality was mentioned during training sessions.

Participants were invited to participate in a semi-structured interview every six months after implementation of the intervention. Interviews focused mainly on quantitative outcomes and included a battery of questionnaires to assess issues such as health status, quality of life, social service use, and perceptions of technology. Some open-ended questions were included in the interviews to allow participants to expand on various issues (e.g., perceptions of technology). Data from these interviews suggested that clients benefited from the use of LSR by having a reduced number of psychiatric hospitalizations, outpatient visits, arrests, and increased community integration. Details of implementation of the intervention, adoption by clients and care providers, and study outcomes have been reported elsewhere [8–11].

### The present study

In addition to semi-structured interviews, clients were able to participate in focus groups throughout the study; focus group discussions centered on issues such as usability, ethical use issues, and recommendations for improvement of the intervention. This approach to data collection allows for the development of innovative health technologies with a focus on user-centered design. User-centered design highlights the importance of usefulness, ease of use, and acceptance by individuals of information technologies [29–31]. As such, the present study reports a qualitative analysis of an electronic PHR used by individuals with a mood or psychotic disorder with data collected during client focus group sessions. This analysis will elaborate upon quantitative data presented elsewhere [8], and provide a more comprehensive understanding of clients’ experiences using the LSR and smartphone (i.e., the MHEN technologies).

### Research questions

Qualitative analysis of MHEN focus group data addressed several questions:

1. What are facilitators to client adoption of the MHEN Technologies?
2. What are barriers to client adoption of the MHEN Technologies?
3. What are the outcomes for clients using the MHEN Technologies?
4. What recommendations do clients have for future development and implementation?

## Methods

### Design

The MHEN was a mixed methods study that employed a delayed implementation design. Client participants were randomized into either the early intervention group (Group A) or the delayed intervention group (Group B). Group A participants received the LSR and smartphone first, while Group B participants received the LSR and smartphone six months later, thus, acting as a control group for the first six months of the study. Details of study implementation and outcomes have previously been reported [8–11]. In addition to completing semi-structured individual interviews every six months, client participants from both Group A and Group B were able to volunteer for focus groups which occurred at three time points throughout the study: 1. within a month of receiving the intervention, 2. at 4–6 months post-intervention, and 3. at 7–8 months post-intervention. Because the themes arising from both Groups A and B were the same, the groups are collapsed in the findings. The duration of focus groups was approximately one hour. Participants provided informed consent in writing prior to participation in the study and prior to each focus group. The research team received ethics approval from Western University in December 2011.

### Sample

Four hundred individuals experiencing a mood or psychotic disorder were recruited from the caseloads of 54 mental health care providers in London, Ontario and the surrounding area to participate in the MHEN study. Mental health care providers were recruited from a number of community mental health teams with the purpose of obtaining a broad representation of community mental health staff. Each team was asked to provide three staff volunteers. Care providers identified potential participants and assessed their interest in participating in the study. Interested clients contacted the research team directly to indicate their interest and schedule a time for registration. The research team then assessed whether the individual met the criteria for being included in the study and obtained written consent. In the end, only seven individuals chose not to participate, although their reasons for this were not given.

Participants were between the ages of 18 and 80 and were clients of London Health Sciences Centre, St. Joseph’s Health Care (London and St. Thomas), the Canadian Mental Health Association, and WOTCH Community Mental Health Care Services. As a result of dropouts and loss of contact with participants, 394 individuals completed the study. A total of 95 participants engaged in focus groups throughout the study (see Table 1). Study participants were eligible to attend more than one of the 18 focus group sessions; the total number of unique participants is not known as identifying information was not collected at focus group sessions and was eliminated from focus group transcriptions.

**Table 1:** Focus group attendance

Time point	Male attendees (n)	Female attendees (n)	Total attendees (n)
Within a month of receiving intervention	14	7	21
4–6 months post-intervention	16	13	29
7–8 months post-intervention	26	19	45
Total	56	39	95

### Data collection

Client focus group discussions were led by experienced mental health clinicians (i.e., a psychiatrist or specialist in clinical mental health nursing), however, individuals participating in the focus groups had not previously interacted with these clinicians in any clinical capacity. Discussions focused on facilitators and barriers for use and adoption of the technologies, outcomes, recommendations for improvement, and other ethical considerations. Focus group facilitators prompted participants with a series of questions including, “What is your experience of using the personal health record?”, “What is your experience of using a smartphone to access the data?”, “How was your care using the personal health record as compared to usual care?”, and “Do you have any privacy or confidentiality concerns?” (see Table 2 for a full list of questions). Focus group discussions were audio recorded and later transcribed verbatim and validated. Throughout the focus group process, anonymity and confidentiality were maintained. Although some information on participants was recorded (i.e. estimated age range, gender), names were not used or recorded in any manner.

**Table 2:** Focus group questions used by facilitators to guide discussion

Time Point	Questions
Within a month of receiving intervention	
	1. What is your experience of using the LSR?
	2. What is your experience of using a smartphone to access the data?
	3. How was your care using the LSR as compared to usual care?
	4. What issues were encountered?
	5. How did the technology assist?
	6. What obstacles exist?
	7. Have you noticed any health improvements since adopting the new technology?
	8. Are there any additional pieces of information you would like to store on your LSR?
	9. Do you have any privacy or confidentiality concerns
	10. What has gone well with the intervention? What can be improved?
	11. What other issues regarding the MHEN project would you like to share?
4–6 months post-intervention	
	1. What is your experience of using the LSR?
	2. What is your experience of using a smartphone to access the data?
	3. How was your care using the LSR as compared to usual care? Changing relationship with care provider?
	4. How did the technology assist?
	5. Have you found any health improvements since adopting the new technology? Other changes? Unanticipated benefits or problems?
	6. What obstacles exist?
	7. Are there any additional pieces of information you would like to store on your LSR?
	8. Is there other information you access using the phone?
	9. What are issues related to maintenance or loss?
	10. How has this changed over time? Is there fall off of use?
	11. Do you have any privacy of confidentiality concerns
	12. What strategies encourage people to continue to use the technology?
	13. Is it worth the money and effort?
	14. What are concerns regarding the end of the project?
7–8 months post-intervention	
	1. What is the difference now compared to earlier in the project?
	2. How else do you connect with people?
	a. Do you have access to a phone at home?
	b. Do you have access to a computer with Internet access at home?
	c. Did you have a personal mobile phone (i.e., not a smartphone) prior to starting this project?
	d. Did you have a personal smartphone (i.e., with data plan and web access) prior to starting this project? If so please indicate make and model.
	e. Nature of prior phone plan.
	3. What is the value of this solution? Base on my experience with this project, if required, what is the maximum you would be willing to pay to continue the program (including device, data plan, clinician support, etc.)

### Analysis

Focus groups were analyzed by three researchers independently using Leininger’s phases of qualitative data analysis [32]. Once focus group transcripts were developed and validated, research team members determined and validated a coding structure for the data. Descriptors occurring repeatedly were analyzed to assess similarities or differences in meanings. During the next phase of analysis, recurrent findings were identified. These recurrent findings were then synthesized into unique concepts. The data were analyzed until saturation occurred, meaning that no novel themes emerged [32]. This occurred at the final data collection point with no new themes being identified with these final focus groups. A matrix approach was used, with themes separated by time of focus group [33]. Triangulation of the themes identified by the individual researchers occurred and all themes were agreed upon. Sub-themes and salient illustrative examples were identified in order to further elaborate on predominant concepts.

## Results

Qualitative analysis of discussions that occurred during 18 focus group sessions revealed important concepts related to each of the four research questions regarding facilitators to use, barriers to use, outcomes of use, and recommendations for improvement. Emergent themes were similar across time points and so these categories have been collapsed. See Table 3 for a summary of overarching themes as well as specific sub-themes describing client perceptions of the MHEN technologies. Illustrative client quotes are provided below to support the qualitative analysis.

**Table 3:** Emergent themes, sub-themes, and illustrative quotes from focus group discussions

Themes	Sub-themes	Illustrative quotes
1. Versatile functionality of the LSR and smartphone facilitated use	▪ Enhanced ability to contact and be contacted (by care providers and family/friends) was important	▪ “…good way for me to…keep in contact with [my]social worker so we could send…messages…in between appointments”
	▪ Tracking and appointment reminders were valuable functions	▪ “…keeps me on track…to actually stay on my meds”
	▪ Applications associated with the smartphone (e.g., music, internet browsing, social media), made it an appealing tool to integrate into daily life	▪ “Definitely I’ve been happier just because I can like actually listen to my music…and watch it…before I could only listen to it, now I can watch it”
2. Aspects of the technologies as barriers to use	▪ Lack of knowledge about technology and data plans prevented use	▪ “I just don’t understand plans, so I just don’t get it. Like the minutes and the data, I don’t get that”
	▪ An onerous login process and the requirement to remember a password for the LSR was challenging	
		▪ “… it was…frustrating to have to…log in at every single point… and…wait for the loading process…”
	▪ The LSR was too slow	
	▪ Prompts and reminders did not always occur as programmed	▪ “I find it sometimes hard to keep track of all the password (sic)I have now”
	▪ Small font and button size on the smartphone was difficult to see/use	▪ “I put it in for a reminder and it emails at the beginning of the day. Well that doesn’t help remind…to take my bedtime meds”
	▪ Smartphone battery life was insufficient	▪ “the text messages are a little too small”
		▪ “the iPhone keyboard is terrible……a real deterrent to using the iPhone”
		▪ “…the battery only lasted a couple hours”
3. Use of the MHEN technologies resulted in perceived positive outcomes	▪ Enhanced feelings of safety, security, independence, and confidence	▪ “I’ve added emergency contacts”
		▪ “I feel more secure because it has all these records and all these things on it, and if I get too depressed I know I can grab my phone for help”
	▪ Increased connection with family/friends and care providers	
	▪ Increased tracking of symptoms and moods	▪ “I’ve enjoyed the instant connection to people”
	▪ Positive effects on mood	▪ “it’s very nice to go back and look at what you’ve written before and also sort of monitor your mood that way”
	▪ Positive feelings towards the MHEN technologies increased over time	
		▪ “I’m more aware of how my moods are, since that I’ve been keeping track of it”
		▪ “I find it really helpful and it is really good to have it in my hand because then I don’t have to go and log on the computer and wait for it to boot up”
4. Future enhancement of the LSR and intervention is recommended	▪ The appearance and functionality of the LSR should be improved	▪ “I would make it [look] less clinical”
		▪ “could be nicer if gives forwarding messages to my email then I [would] get to see it directly”
	▪ Alerts should be sent directly to the smartphone when messages or changes are made to the LSR	
		▪ “there’s so much on the SMART record that I don’t even know what to do with it”
	▪ Additional training should be provided on the use of the LSR	

### Versatile functionality of the LSR and smartphone facilitated use

A variety of factors encouraged study participants to engage with the MHEN technologies. The ability to stay connected with family, friends, and care providers commonly encouraged individuals to use the smartphone and LSR. Participants felt that they could, “*get help faster because it’s with* [them] *all the time.”* Particularly, clients suggested that both the appointment reminders and tracking functions within the LSR were useful, such as when one client suggested that the LSR had *“been amazing for, like, making sure that I get to my counselling appointments…”*, and another said, *“…it’s not just one thing we’re charting, we’re charting our lives… and it’s important.”* Participants also commonly described the value of having the smartphone over and above the usefulness of the LSR. Many smartphone functions, including Internet access, music, and social media were described positively and allowed participants to customize the device to their own interests and needs. For example, one participant said that Facebook was useful in order to, *“keep in touch with family and friends* [because] *otherwise it would be a long distance call*.” Thus, a multi-functional device that allows for greater connection with other people is important for integrating mental health technologies into daily life.

### Aspects of the technologies as barriers to use

Several barriers to using the MHEN technologies were identified throughout focus group discussions. Initially participants expressed fears and frustrations as a result of not fully understanding the technologies. A lack of experience with similar technologies created worries about doing something wrong or going over data allowances. One participant admitted to being, *“terrified to touch* [the smartphone] *because it is so sensitive,”* and another said that, *“there’s so much on the* [LSR] *that I don’t even know what to do with it.”* However, with orientation, increased use, and familiarity with the technology, many of these fears subsided. Some aspects of the LSR itself acted as deterrents to clients, including its operational speed and an onerous login. The LSR was consistently described as *“really slow”*, and participants were dissatisfied with the need to, *“log in at every single point in time and… wait for the loading process.”* These issues were exacerbated by the need to remember passwords, which was challenging for some individuals: *“*[It’s] *hard to keep track of all the passwords I have now.”* Participants also struggled with differences in the client and care provider views of the LSR. Since the two views were not identical, this often created challenges when care providers were attempting to assist clients in using the LSR. Further, some participants found flaws in the prompts and reminders system: *“I would enter times for medication and instead of sending one email it would send me three or four. So, if you’re taking medication a couple times a day, that’s almost a dozen emails… I stopped using that part of the* [LSR]”; *“I put it in for a reminder and it emails at the beginning of the day. Well, that doesn’t help remind…* [me] *to take my bedtime meds.”*

Similarly, the smartphone was challenging for some clients to use. One participant noted that, *“anything that requires written information, it’s easier on the computer because the iPhone keyboard is terrible…it’s a real deterrent to using the iPhone to access the service.”* Another common concern was the limited battery life of the smartphone: *“I find I have to charge my phone all the time.”*

### Use of the MHEN technologies resulted in perceived positive outcomes

Importantly, participants described many positive outcomes of using the MHEN technologies. Individuals noted that they felt safer or more secure both in regards to their physical safety and in terms of receiving support for mental health issues quickly and easily; for example, *“I feel more secure because it has all these records and all these things on it, and if I get too depressed I know I can grab my phone for help.”* Again, participants described feeling more connected to family, friends, and care providers: *“I think the whole project … has changed my entire life… it’s given me more of a social life…I’ve certainly gained a lot more friends and company …you can communicate with them a lot easier and it’s just nice to have that connection.”* One participant said that, *“it keeps me in contact with my mental health professionals out in the community, so it helps* [us to] *communicate when there is no physical presence.”* This increased connection also allowed for proactive engagement of care providers: *“Times where I have been… going downhill, things are spiraling out of control… I’ve put in my mood and my sleep and my meds and that kind of thing, and … my case worker has called me and said, ‘Hey, I see things are going downhill on here, what’s going on? Come and see me.’”*

Participants also described many physical and mental health benefits including assistance with smoking cessation and enhanced self-awareness due to mood tracking. Many participants echoed the sentiment that, *“it’s helping me be more self-aware of my mood swings, my changes and things like that.”* Participants suggested that they became more comfortable with the technologies over time, with some participants expressing initial dissatisfaction using the smartphone: *“If you’d ask me in the first month I would have given it back.”* However, many participants suggested that, *“…the longer I have the phone the better use I make out of it because, um, there’s some things I never used to do with it* [when] *I first got it because I didn’t know how and I thought I might screw it up if I try. But now I’m realizing you can’t really screw it up.”* Finally, participants said that they were happy just to have a mobile phone: *“I get a little glow, it’s like, ‘oh cool, I got a nice phone,’ you know? Like, not a lot of people can afford to have a phone like this. I’m pretty lucky”*; *“It makes me feel, you know, part of the outside world”*; *“Well, I can now say I can use a cell phone, and it feels good.”*

### Future enhancement of the LSR and intervention is recommended

Focus group discussions provided valuable suggestions for improvements to the LSR. Many participants indicated that the functionality of the software could be improved. For instance, greater compatibility across web browsers, simplification, as well as more comprehensive records for medical information were all suggested as possible improvements. Further, participants suggested that it would be useful to receive some information by text or email, so that they, *“get to see it directly”*, rather than having to login to the LSR. Based on initial focus group discussions with Group A participants, some changes were made to the LSR to allow appointments to be pushed directly to the smartphone’s calendar, prior to Group B receiving the intervention.

Some participants also thought that the LSR could be, *“more user friendly towards those… that aren’t computer literate.”* While drop-in sessions were offered for participants who were experiencing difficulties with the technologies, some individuals may have benefitted from additional training or information that was tailored to their level of experience with mobile and Internet technologies: *“I do not think it was explained properly—how to use the phone. I think that you need a lot, to just try to struggle with it yourself….if you don’t know how to use one, it’s very difficult.”*

## Discussion

The lives of individuals living in the community with a mood or psychotic disorder were positively impacted by receiving a web accessible personal health record and a smartphone (i.e., the MHEN technologies) [8, 9]. Qualitative findings from focus group discussions with study participants supported the positive findings from previous quantitative analyses. Due to the clients’ ability to successfully engage with the MHEN technologies, these findings speak to the feasibility of a new and innovative model of mental health care in which the use of technology will be relied upon to increase efficiency in the healthcare system and improve quality of care for clients living in the community.

These qualitative data provide a valuable contribution to the understanding of how smart technologies can be integrated into usual mental health care. Smartphones are extremely portable and commonplace in society. Therefore, clients can use these devices to manage and track mental health issues in any place at almost any time without feeling stigmatized [27]. This is aligned with the many facilitators and positive outcomes of using the MHEN technologies described by participants, supporting quantitative findings from the MHEN study [8]. Specifically, participants experienced significantly increased quality of life as measured by the Lehman Quality of Life Interview [34]. The MHEN technologies not only encouraged participants to manage their health, but also facilitated engagement in enjoyable activities (e.g., listening to music) and maintenance of social support systems through calling, texting, and social media; thus, it is unsurprising that participants experienced an enhanced quality of life. Furthermore, the value of having the smartphone, beyond the use of the LSR (e.g., connecting with family and friends through texting and social media), supports adoption and sustained use of these technologies by clients. The increased ability of individuals to monitor and improve their health, supports the finding that participants in the MHEN study experienced fewer psychiatric hospitalizations and fewer outpatient appointments [8]. Previously presented quantitative findings indicate that mobile and web-based technologies have the capability to significantly improve mental health outcomes for individuals experiencing mood or psychotic disorders. Yet, in order for mobile technologies to be integrated into usual care, it is important that clients subjectively perceive that the intervention is improving their well-being, and so positive client reports from focus group discussions provide essential support for the integration of technology into mental health care.

For successful implementation of mental health smart technologies, in addition to knowing what works well, it is important to understand what needs to be improved. Participants expressed several frustrations about inherent aspects of the smartphone and LSR. For example, some individuals identified that the text size and touch screen keyboard made the intervention difficult to use. Future interventions may benefit from allowing a choice of devices to ensure accessibility for all participants. Some participants experienced difficulties remembering passwords or were frustrated by the login process. Previous studies have identified that cognitive impairments can impede one’s ability to use technology [35]. Since the MHEN sample included individuals with a wide range of illness severity and impairments, it is possible that certain aspects of the LSR were too cognitively taxing for some participants. Alternatively, difficulty in remembering passwords may be a function of the pervasiveness of mobile devices and applications requiring passwords, rather than a specific cognitive deficit. Therefore, including a secure login process that does not require the user to remember a password, such as biometric identification, may enhance the usability of the LSR for a greater number of people. Further, participants suggested that receiving some information by text or email, such as when a message from a care provider is received, rather than having to login to the LSR, would be useful. These recommendations are consistent with previous research describing how electronic information should best be displayed for individuals with mental illnesses [36], and will inform future development of mobile and web-based technologies for mental health clients.

### Limitations

A notable limitation of this study is the difficulty in separating client perceptions of the hardware (smartphone) and the software (LSR). Many participants described that simply having a smartphone, or even a phone, positively impacted their lives, and so perceptions of the LSR may be skewed by positive perceptions of the smartphone. Conversely, some individuals had difficulty using aspects of the smartphone, such as the touchscreen keyboard, and so usability of the LSR may have been negatively impacted by the device itself. Additionally, the care provider and client views of the LSR were not structured identically, creating difficulties when care providers and clients were attempting to navigate the system together. However, this inconsistency can easily be addressed in future software development. The research team was also not able to assess whether participants were accessing the desktop version of the LSR or the mobile version of the LSR from their mobile devices; while it was possible to see the number of “hits” to the desktop and mobile versions of the LSR, if clients accessed the desktop version using their smartphones, this would have been recorded as a “hit” to the desktop version. Perceptions of the LSR may have varied depending on the preferred method of access, and so despite the inherent difficulty, future interventions should attempt to tease apart perceptions of distinct aspects of the intervention.

Another possible limitation is that only individuals who wanted to use technology in their care would have agreed to participate in the study. This may have skewed the findings towards viewing the MHEN technologies positively. However, this limitation is mitigated by the fact that in regular clinical care, the use of technology would not be imposed on any clients who do not perceive it as a desirable augmentation of care. Additionally, the sample used in this study was broad, but did not specifically investigate the use of technology in sub-populations, such as the very young or the very old. Each of these populations may have specific technological needs; for example, younger individuals who are more comfortable with technology may desire more complex or customizable functionality, while older adults, who may be unfamiliar with technology, may require a simpler format or more extensive training.

### Implications

This qualitative analysis provides evidence that, in addition to quantifiable benefits, clients view the MHEN technologies as useful for improving mental health. These findings are particularly significant when considering that the scope of the MHEN project is much larger, in terms of number of participants and duration of study, than many other similar studies in the literature [26, 28]. This additional support for the usefulness of mental health smart technologies has the potential to influence funding models and the development of future interventions. Support for the usefulness and acceptability of mental health smart technologies may encourage investment in adopting similar interventions into usual care. Further, this research will assist in optimizing mobile and web-based interventions for use by mental health clients. Future interventions should aim to parse out the different influences of phones, smartphones, and using an electronic personal health record solely on a desktop computer, as well as investigate the needs of specific sub-populations, such as young people or older adults.

## Conclusions

The client’s perspective is of vital importance to understanding the effectiveness and likelihood of uptake of mental health mobile and web-based technologies. This qualitative data demonstrates the potential benefit of using mobile phones and web applications in mental health care. However, future research should seek to improve software functionality and disentangle hardware and software preferences to provide a better understanding of mental health smart technologies.

## Abbreviations

LSR: Lawson SMART Record
MHEN: Mental Health Engagement Network
PHR: Personal health record
